# The downregulation of hormone-sensitive lipase and dysregulation of cholesterol receptors/transporter affect testicular lipid homeostasis and function in HFD-induced oligoasthenospermia mice

**DOI:** 10.1186/s10020-025-01327-x

**Published:** 2025-08-04

**Authors:** Min Pan, Jingya Li, Yujia Wang, Ziao Liu, Li Li, Tongsheng Wang

**Affiliations:** 1 Pharmacological Department of the College of Integrated Chinese and Western Medicine, Anhui University of Chinese Medicine, No 350 Longzihu Road, Hefei, 230012 China; 2 Basic Medical College, Hebei Medical University, Shijiazhuang, China

**Keywords:** Obesity with oligoasthenospermia, Lipid homeostasis, HSL, Cholesterol receptors, Cholesterol transporter, Testicular function

## Abstract

**Background:**

Obesity-induced oligoasthenospermia is associated with testicular lipid metabolism. However, the mechanisms underlying the lipid homeostasis imbalance of obesity-induced oligoasthenospermia are unclear.

**Methods:**

Male C57BL/6 mice fed a high-fat diet were established for the in vivo model. TM3/TM4 cells were treated with palmitic acid (PA) in vitro. Proteomics analyzed differential proteins in the testis. The Oil red O and Nile red were used to observe lipid droplets (LDs). Filipin staining was used to observe free cholesterol (FC). Hormone-sensitive lipase (HSL), Low-density lipoprotein receptor (LDLr), Scavenger receptor class B type I (SR-BI), and ATP-binding cassette transporter A1 (ABCA1) expressions were analyzed using qRT-PCR, WB, Immunofluorescence, and immunohistochemistry. Testosterone synthesis and Blood-testis barrier (BTB) integrity were evaluated by ELISA, Transmission electron microscope, and WB.

**Results:**

HFD mice exhibited elevated blood lipid, reduced sperm quality, and hormonal imbalances. Meanwhile, testosterone synthesis was impaired, and BTB was damaged in HFD mice. In vivo and in vitro models, LDs deposition was observed, and HSL expression was down-regulated in Leydig and Sertoli cells. However, the expressions of cholesterol uptake receptors and efflux transporters, as well as the levels of FC, in the two cells were inconsistent. In Leydig cells, the expression of cholesterol uptake receptors (LDLr and SR-BI) was upregulated, resulting in increased FC levels. In Sertoli cells, cholesterol efflux transporter (ABCA1) expression was upregulated, and FC levels decreased. Overexpression of HSL ameliorated LDs accumulation and increased testosterone levels.

**Conclusion:**

The down-regulation of HSL and dysregulation of cholesterol receptors/transporter may affect lipid homeostasis, thereby damaging testicular function.

**Supplementary Information:**

The online version contains supplementary material available at 10.1186/s10020-025-01327-x.

## Introduction

Obesity is a chronic, recurrent, and progressive disease that has become a global health problem. In the past 50 years, obesity has been increasing, and the global obesity prevalence rate has doubled for women and more than tripled for men. High-energy food, high sugar intake, and reduced physical activity are the main driving factors for obesity (Chen et al. 2023). Studies have shown that a percentage of male body fat exceeding 25% increases the risk of obesity complications (Adler et al. 2013). Obesity is not only related to diabetes, cardiovascular disease, cancer, asthma, sleep disorders, and increased risk of all-cause death but also affects male reproduction (Leisegang et al. 2021). Specifically, an increase in the plasma cholesterol level is accompanied by damage to the blood-testis barrier (BTB), which leads to spermatogenesis disorders. Obesity is closely related to lower sperm volume, density, and vitality and a higher rate of sperm head deformity. Obesity can affect testosterone levels in men. The higher the BMI is, the lower the testosterone level (Peel et al. 2023; Ameratunga et al. 2023). According to the WHO Laboratory Manual for Human Semen Examination and Processing (6th Edition), the diagnosis of oligoasthenospermia must meet two or all of the following three criteria: the sperm concentration < 16 × 10⁶/mL, progressivesperm motility (PR%) < 30%, and the normal morphology sperm (%) < 4% (Wang et al. 2022). Therefore, obese men are more likely to suffer from oligozoospermia and asthenospermia than men with a normal weight.

In testis, the lipid is the "fuel" for cells, which is very important for spermatogenesis (Du et al. 2023). Cholesterol is not only an important substrate for androgen synthesis, but also a key component for maintaining the structure and function of cell membranes. However, several studies have shown that excessive cholesterol is toxic. Excessive cholesterol accumulation in the testis may cause testosterone deficiency (Song et al. 2021). Excess cholesterol in cells is esterified to form cholesterol esters, which then form lipid droplets (LDs) with other neutral lipids (Feingold 2022; Dekkers et al. 2023). Excessive LDs may change the cytoskeleton when lipid droplets are excessively deposited in cells. Studies have shown that the accumulation of LDs in Sertoli cells will destroy the BTB (Ge et al. 2022; Shah et al. 2023). Therefore, the balance of testicular lipid homeostasis is critical to maintain the normal reproductive function of men.

Steroid production or spermatogenesis requires cholesterol, and the cholesterol synthesised by itself is not enough to maintain cell function. Therefore, it is necessary to rely on the intake of exogenous cholesterol: (1) Receptor-mediated lipoprotein-derived cholesterol uptake. (2) Neutral cholesterol ester hydrolases (Hormone-sensitive lipases, HSL) hydrolyze cholesterol esters stored in LDs (Luo et al. 2020; Wang et al. 2014). The two main mechanisms of lipoprotein cholesterol uptake are the low-density lipoprotein receptor (LDLr) and Scavenger receptor class B type I (SR-BI). LDLr ingests lipoprotein-cholesterol particles through endocytosis, and the channel formed by SR-BI dimerisation transports cholesterol esters located at the lipoprotein core into the cell (Luo et al. 2020; Ikonen and Olkkonen 2023). Internalised cholesterol esters are stored in lipid droplets and hydrolysed by cholesterol esterase to generate free cholesterol, which is utilised in cellular processes such as steroid production or maintaining membrane integrity (Olzmann and Carvalho 2019). In testis, HSL is the only enzyme that can hydrolyze cholesterol ester. The loss of HSL activity will lead to the accumulation of cholesterol esters and diacylglycerol, thereby altering cholesterol homeostasis. Converting cholesterol ester into free cholesterol through HSL is essential in controlling cholesterol in steroid and sperm production (Kraemer and Shen 2002; Osuga et al. 2000). It is found that the purified mice with HSL allele mutation have normal phenotypes but oligozoospermia, which may be due to the inability to hydrolyze cholesterol esters to maintain cholesterol homeostasis (Chung et al. 2001). Therefore, the balance of cholesterol metabolism in the testis is very important. ATP-binding cassette transmembrane transporter mediates the excretion of free cholesterol. ATP-binding cassette transporter A1 (ABCA1) is expressed in testis (Bloise et al. 2016). This indicates that ABCA1 may play a role in lipid transport in the testis. Abnormal cholesterol metabolism has a negative impact on male reproduction, and it is unclear how obesity affects the HSL and cholesterol receptor/transporter in the testis, thereby altering lipid homeostasis.

This study posited that obesity-associated dysregulation of testicular cholesterol metabolism and lipid imbalance constitutes pivotal mechanisms underlying oligoasthenospermia. We employed complementary in vivo and in vitro models, an HFD-fed murine model, and palmitic acid (PA)- treated cell cultures. Systematic analyses were conducted to evaluate three critical dimensions: (1) Lipid regulatory machinery: Expression patterns of hydrolytic enzyme (HSL), cholesterol uptake receptors (LDLR, SR-BI), and efflux transporters (ABCA1); (2) Steroidogenic function: Transcriptional regulation of testosterone biosynthesis enzymes and associated hormonal profiles; (3) Structural integrity: BTB ultrastructure in lipid metabolic perturbation.

## Materials and methods

### Ethics statement

Male C57BL/6 mice were purchased from Hangzhou Ziyuan Experimental Animal Science and Technology Co., Ltd. (production license number: SCXK (Zhejiang) 2019–0004). The experimental protocol was reviewed and approved by the Animal Care and Use Committee of Anhui University of Traditional Chinese Medicine (animal ethics number: AHUCM-mouse-2023003).

### Animals and treatment

Forty-eight male C57 BL/6 mice (8 weeks old) were randomly divided into the control group (n = 24) and the model group (n = 24). The control group was fed with normal diet (ND—10% fat, Huafukang Biology Co., Ltd., 2,410,320,605, Beijing, China), while the model group was fed with high-fat diet (HFD—60% fat; A121956, Xietong Biological Co., Ltd., Jiangsu, China). The experimental period lasted for 12 weeks (age: 8 weeks to 20 weeks). Body weight and food intake were recorded weekly throughout the experiment. At the end of the study, venous blood was collected for the measurement of sex hormone levels and lipid profiles. Following anaesthesia with 1.25% Tribromoethanol (0.2 mL/10 g ip, Nanjing, M2910, Aibei Bio., China), euthanasia was performed. The samples of sperm, epididymis, testes, adipose tissue (epididymal fat and abdominal fat), and liver were collected and preserved for subsequent experiments.

### Body weight and Lee’s index

Each mouse was weighed every 7 days during the experiment. At the end of the experiment, the mice were narcotized with 1% pentobarbital, and their body length (from nasal to anal length) was measured. Lee’s index was calculated to evaluate the degree of obesity. Lee's index = [(Body weight)^(1/3)]/Body Length.

### Sperm quality

The epididymis was cut in 1 mL of saline at 37 °C. After being incubated for 10 min, a drop of the sperm filtrate was placed on a Makler sperm counting chamber (Makler, Sefi-Medical Instruments, Ltd., Israel) and observed under a microscope. The total number of sperm was calculated, and the sperm quality was rated. The sperm observed via microscopy were rated according to the following criteria: (a) rapid progressive motility, (b) slow or sluggish progressive motility, (c) nonprogressive motility, and (d) no motility. The formula for calculating sperm quality was as follows: sperm density = (a + b + c + d) × 10^6^/mL; sperm motility = [(a + b)/(a + b + c + d)] × 100%; sperm survival rate = [(a + b + c)/(a + b + c + d)] × 100%; Class A sperm = [a/(a + b + c + d)] × 100%.

### Hormone levels and biochemical indices

The levels of leptin (Meimian, MM-0622M2, Jiangsu, China), testosterone (T), luteinizing hormone (LH), follicle-stimulating hormone (FSH) and inhibin-B (INHB) (Jianglai, JL25196, JL10432, JL10239, JL10989, Shanghai, China) in serum were detected via ELISA kits according to the manufacturer’s instructions. The levels of biochemical markers in serum, including triglycerides (TG), total cholesterol (TC), low-density lipoprotein cholesterol (LDL-C), and high-density lipoprotein cholesterol (HDL-C) (Jiancheng, A111-2-1, A110-1-1, A113-1-1, A112-1-1, Jiangsu, China) were detected according to the kit instructions.

### The level of testosterone in testis, serum and TM3 cell

Testicular tissue was homogenised in pre-cooling PBS (1:9 w/v ratio), then centrifuged at 5000 × g for 10 min at 4 °C. The resulting supernatant was collected. According to the manufacturer’s instructions, the level of testosterone was detected by Elisa kit (Meimian, MM-0569M1, Jiangsu, China).

The collected whole blood samples from mice were left at room temperature for 2 h, then centrifuged at 1000 × *g* for 20 min, and the serum was collected. The culture supernatant of TM3 cells was collected and centrifuged at 1000 × *g* for 20 min. The resulting supernatant was then collected for T analysis. The levels of T (Jianglai, JL25196, Shanghai, China) were detected via ELISA kits according to the manufacturer’s instructions.

### HE staining

Livers and testes were dehydrated, embedded in paraffin, sectioned into 6 μm slices, and stained with hematoxylin and eosin. Tissue sections were observed and taken under a slide scanner (WISLEAP, WS-10, Beijing, China).

### Sperm morphology

A sperm smear was prepared via the push‒slide method. The mixture was fixed in methanol for 5 min and dried naturally. The smear was stained with 2% eosin solution for 20 min, then rinsed with distilled water and allowed to dry naturally. The morphology of the sperm was photographed with a slide scanner, and the abnormality rate was calculated. The abnormality rate (%) = abnormal sperm number/500 × 100%.

### Oil red O and Nile red staining

The testicular tissue embedded in optimal cutting temperature (OCT) compound was frozen and sliced (thickness: 10 μm) using a cryostat (Leica, Germany). The tissue was then fixed in 4% paraformaldehyde for 10 min and subsequently washed with PBS. After drying, the slices were stained with Oil red O (Servicebio, G1015, Wuhan, China) for 20 min. Then, the cells were differentiated with 60% isopropanol for 15 s. Tissue sections were taken with a slide scanner. Similarly, after the frozen tissue sections were fixed with 4% paraformaldehyde, the sections were incubated with Nil red staining solution for 15 min at room temperature in the dark and observed under an immunofluorescence microscope. For the cell experiments, after the cells were removed and fixed at room temperature for 10 min, the operation and observation steps for Oil red O and Nile red staining were the same as above.

### Filipin staining

Filipin staining analysis was conducted to examine free cholesterol in the testis or cells. Testicular sections were fixed in 4% paraformaldehyde for 10 min, after which the samples were washed with PBS. Filipin dye solution (GLPBIO, GC12048, California, USA) was added, and the samples were dyed in the dark for 20 min at room temperature. The samples were observed under a fluorescence microscope (Leica, Dmi8, Germany) and quantified by a Fluorescence Microplate Reader (Molecular Devices, Gemini XPS, USA).

### Transmission electron microscopy (TEM)

Testicular tissue was cut into 1 cm^3^ pieces and fixed in 2.5% glutaraldehyde for 2 h at room temperature. Then, the tissue was rinsed in 0.1 M PBS and fixed in 1% osmium tetroxide for 2 h. The sample was dehydrated by processing it in a graded series of acetone washes. The samples were soaked in epoxy resin for 2–3 h, embedded, heated to 70 °C in an oven for 30 min, and embedded with Epon 812 epoxy resin in predried moulds. The individual resin monomers (epon) formed a hard plastic and were placed in an oven at 40 °C for 12 h and 60 °C for 48 h. After trimming the embedded block, it was ultra-thinly sectioned to 70 nm, fished with copper mesh, and stained with electrons (lead and uranium). The samples were observed and photographed under an H-600 transmission electron microscope (Hitachi Electronics, Japan).

### Proteomics

The testis tissue was ground with liquid nitrogen into a cell powder. After that, four volumes of lysis buffer (8 M urea, 1% protease inhibitor cocktail) were added to the cell powder, followed by sonication for three min on ice via a high-intensity ultrasonic processor (Scientz). The remaining debris was removed by centrifugation at 12,000 rpm at 4 °C for 10 min. Finally, the supernatant was collected. Trypsin was added at a 1:50 trypsin-to-protein mass ratio overnight. Peptides were dissolved in mobile phase A of liquid chromatography and separated by a NanoElute ultrahigh-performance liquid system. The peptides were subjected to capillary source chromatography and time-of-flight Pro mass spectrometry.

#### Immunohistochemistry

According to the instructions of the immunohistochemical two-step kit (ZGB-Bio, PV-9000, Beijing, China), the testicular tissues were embedded in paraffin and sliced into 6-μm sections. The samples were boiled in sodium citrate buffer (0.01 M, pH 6.0; Leagene, Beijing, China) for 20 min for antigen recovery. After cooling to room temperature, the slices were incubated in reagent 1 for 10 min at room temperature to block endogenous peroxide activity. The samples were incubated with an HSL antibody (1:200; Immunoway, #YT2240, Beijing, China) at 4 °C overnight. Reagent 2 (reaction enhancer) was added dropwise, and the mixture was incubated at 37 °C for 20 min. Then, the goat anti-mouse/rabbit IgG polymer labelled with enhanced enzyme was added dropwise and incubated at 37 °C for 20 min. DAB chromogenic solution (ZGB-Bio, ZLI-9019, Beijing, China) was added, and the samples were incubated for approximately 5 min. Then, the nuclei were stained with hematoxylin and sealed with neutral resin. The distribution and expression of HSL were observed via a slide scanner.

### Immunofluorescence

The testicular tissue sections were deparaffinized and blocked with 3% bovine serum albumin. The sections were then incubated with anti-ABCA1 (1:50; Immunoway, #YN2847; Beijing, China) or anti-ZO-1 (1:100; Servicebio, GB111402; Wuhan, China) antibodies at 4 °C overnight. Next, Alexa Fluor® 488-labelled goat anti-rabbit secondary antibody (1:200, ZSGB-BIO, ZF-0511, Beijing, China) was incubated at room temperature for 1 h. Antifade Mounting Medium with DAPI was added to sections. The fluorescence intensity was observed and photographed under a fluorescence microscope (Leica, Dmi8, Germany).

### Cell cultivation and treatment

Mouse Leydig cell TM3 cells were obtained from the Institute of Basic Medicine, China Academy of Medical Sciences (Beijing, China), and mouse Sertoli cell TM4 cells were purchased from Procell Life Science and Technology Co., Ltd. (Wuhan, China). Both TM3 and TM4 cells were cultured in DMEM-F12 containing 5% horse serum, 2.5% fetal bovine serum, 1% NEAA, and 1% penicillin‒streptomycin, and the culture environment was 37 °C and 5% CO_2_. Palmitic acid (PA; Sigma, P0500; Saint Louis, USA) induced lipid deposition in the cell experiments. For testosterone measurement, TM3 cells (1 × 10^5^ cells/well) were seeded in 6-well plates and cultured for 24 h. After adherence, the cells were stimulated with 10 IU/mL hCG (5197 IU/mg; GLPBIO, GP21266, California, USA) for 24 h. Then, different treatments were applied according to the experimental design. Following 24 h of treatment, the culture supernatant was then collected for testosterone quantification (Alavi et al. 2022; Sun et al. 2019).

### Transfection (siRNA and overexpression plasmids)

The plasmid vector *Hsl* and pcDNA3.1 were designed and synthesised by GenePharma (Shanghai, China). siRNA-*Ldlr*, siRNA-*Scarb1*, siRNA-*Abca1*, and negative control (NC) were obtained from GenePharma (Shanghai, China). These sequences are recorded in Supplementary Table 1. According to the manufacturer's instructions, the siRNA or the overexpression plasmid vector was transfected into TM3/TM4 cells. The cells were collected 24–48 h after transfection. The knockdown efficiency and overexpression efficiency were evaluated via qRT-PCR and western blotting.

### qRT‒PCR

Total RNA was extracted from the cells via a SPARKeasy Rapid Extraction Kit (Spark Jade, AC0201, Shangdong, China). According to the manufacturer's instructions, cDNA was synthesized via a reverse transcription kit (BIOS Harper, BL 696a, Shanghai, China). Real-time PCR detection was performed using a LightCycler 96 thermal cycler and qPCR SYBR Green Master Mix (Yeasen, 11201es, Shanghai, China). The primers were synthesised by Shenggong (Shanghai, China), and these sequences can be found in Supplementary Table 2. Each reaction system consisted of 10 μL of SYBR green, 2 μL of cDNA, 0.4 μL of PCR forward primer, 0.4 μL of PCR reverse primer, and 7.2 μL of DEPC-treated water. The thermal cycling conditions included predenaturation at 95 °C for 5 min and 40 amplification cycles. The parameters were as follows: denaturation at 95 °C for 10 s, annealing at 55 °C for 20 s, and extension at 72 °C for 20 s. All the data were homogenised with β-actin as the internal reference gene, and the expression of the target gene was calculated as a multiple change in the 2^−ΔΔCt^ value.

### Western blot

Tissue homogenates or cells were lysed in RIPA lysis buffer containing 1 mM PMSF at 4 °C for 30 min to extract total protein. Total protein levels were determined via a BCA protein detection kit (Spark Jade, EC0001 Shandong, China). The extracted protein was separated via SDS-PAGE, transferred to a PVDF membrane, and sealed with 5% skim milk powder at room temperature for 1–2 h. The membrane was mixed with antibodies against HSL (1:1000), ABCA1 (1:1000), Translocator protein (TSPO; 1:1000) (Immunoway, # YT2240, # YN2847, # YT3612, Beijing, China), LDLR (1:1000), SR-BI (1:1000), N-cadherin (1:1000), β-actin (1:5000) (Zhengneng, R380860, R25674, R380671, R380624, Chengdu, China), 17β-HSD (1:1000), 3β-HSD (1:1000) (Proteintech, 15,116–1-AP, 15,516–1-AP, Wuhan, China), Cx-43 (1:2500), vimentin (1:1000) (Servicebio, GB11234, GB11192, Wuhan, China), and Steroidogenic acute regulatory protein (StAR; 1:2000) (Cusabio, CSB-pa 174,690, Wuhan, China) and incubated at 4 °C overnight. The membrane was subsequently incubated with a secondary antibody (1:10,000; ZENBIO, 511,203, Chengdu, China) for 1 h at room temperature. An enhanced chemiluminescence detection kit (Biosharp, BL520B, Shanghai, China) was used to detect the protein bands, an automatic chemiluminescence image analysis system (Tanon 5200, Shanghai, China) was used to collect images, and the grayscale of each protein band was quantitatively analysed via ImageJ.

### Statistical analysis

Statistical analyses were performed via SPSS 23.0 (SPSS Inc., Chicago, IL, USA) and GraphPad Prism 9.5 software. The results are expressed as the mean ± standard deviation (SD) for at least three different studies. T-test analysis of independent samples was used to evaluate the differences between the two groups. One-way analysis of variance (ANOVA) followed by least significant difference (LSD) analysis was conducted. In cases of uneven variance, the Games–Howell analysis was performed using the Welch ANOVA method. Differences were considered significant when the *p* value was < 0.05.

## Results

### Obesity-induced oligoasthenospermia in male mice

**Fig. 1:**
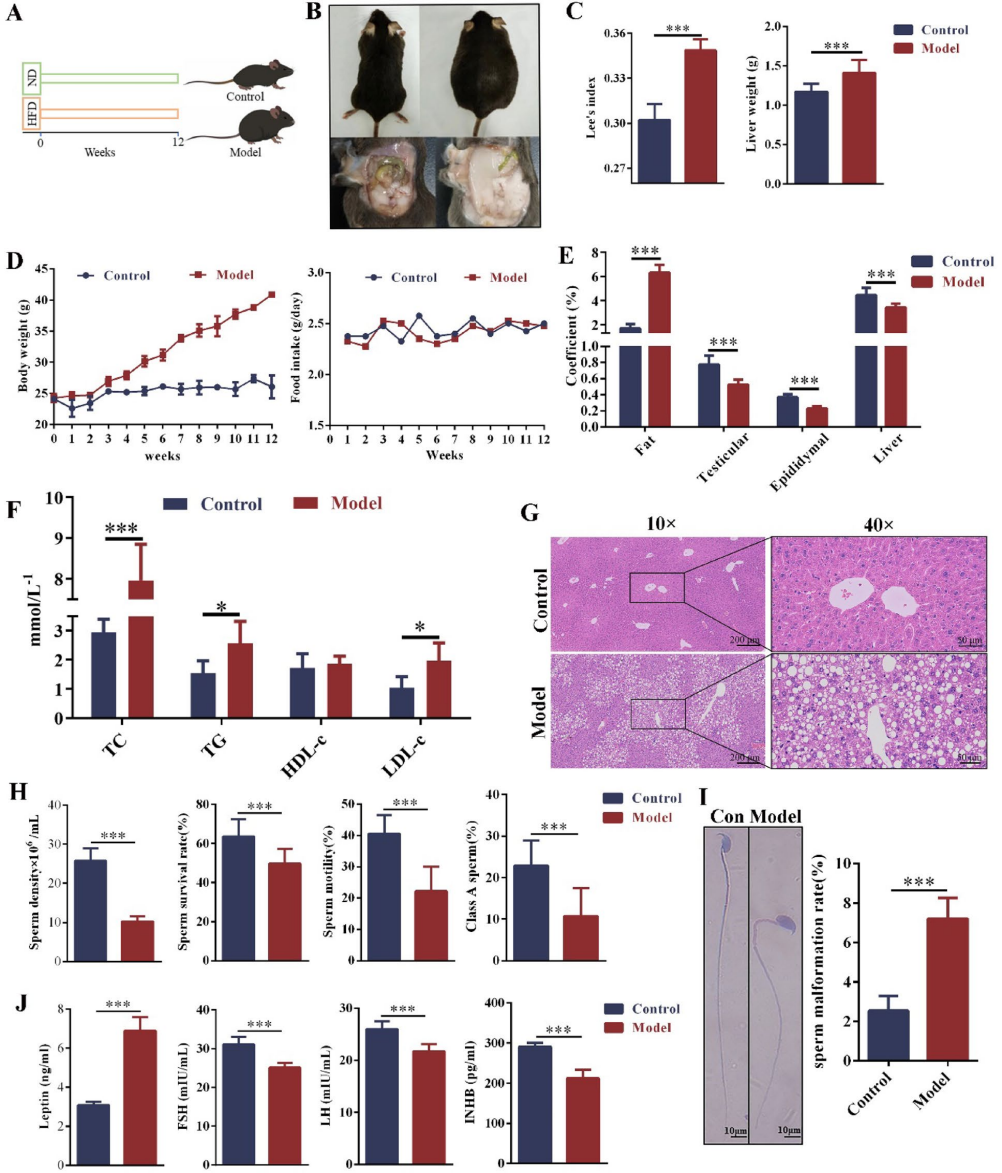
The obesity with oligoasthenospermia model was established via HFD. **A** Mice were fed ND or HFD for 12 weeks. n = 24. **B** Image of back and abdomen. **C** Lee's index and liver weight. n = 24. **D** Body weight and food intake. n = 24. **E** Coefficients of fat and organs (liver, epididymis and testis). n = 24. **F** Blood lipid levels. n = 6. **G** HE staining of the liver (scale bar: 200 μm and 50 μm). n = 3. **H** Sperm density, survival rate, motility and A-level sperm rate. n = 24. **I** Abnormal sperm morphology and sperm malformation rate (scale bar: 10 μm). n = 6. **J** Hormone (Leptin, LH, FSH, and INHB) levels in serum. n = 6. ND = normal diet, HFD = high-fat diet, LH = luteinizing hormone, FSH = follicle stimulating hormone, INHB = inhibin B. Compared with the control group, *p < 0.05, ***p < 0.001

To investigate the effects of HFD-induced obesity on hormone levels and sperm quality in mice, the control group and the model group were fed a standard diet and a high-fat diet for 12 weeks, respectively (Fig. 1A). Compared with the control group, the body weight of the model group mice increased significantly starting from the 4th week. Although food intake fluctuated weekly, there was no significant difference between the two groups (Fig. 1D). Compared with the control group, the model mice exhibited significant fat accumulation in the abdominal and periorgan regions (Fig. 1B). In the model group, Lee's index, liver weight, and fat coefficient were increased significantly (Fig. 1C, E). However, the organ coefficients of the liver, testes, and epididymis were significantly reduced (Fig. 1E). The levels of TC, TG, and LDL-C were significantly elevated, but HDL-c was no significant difference (Fig. 1F). The H&E staining of the liver showed that the control group exhibited normal liver structure, with orderly arranged hepatocyte cords, distinct hepatic lobules and sinusoids. In contrast, the model group displayed disorganised hepatocyte cords, abnormal hepatocyte arrangement, and many vacuoles (Fig. 1G). These results indicated that the obesity model was successfully established in mice. Sperm was collected from the epididymis and observed under a microscope to assess sperm quality. Compared with the control group, sperm density, motility, viability, and class A sperm rate were significantly decreased in the obese mice (Fig. 1H). The sperm malformation rate was significantly increased in the model group, with a higher incidence of round-headed sperm and sperm neck abnormalities (Fig. 1I). In the model group, serum leptin levels were significantly elevated, while FSH, LH, and INHB levels were significantly reduced (Fig. 1J). These results demonstrated that HFD-induced obesity impaired sperm quality and hormone levels, leading to oligoasthenospermia in male mice.

### The lipid droplet deposition in the testes of obese mice with oligoasthenospermia

**Fig. 2:**
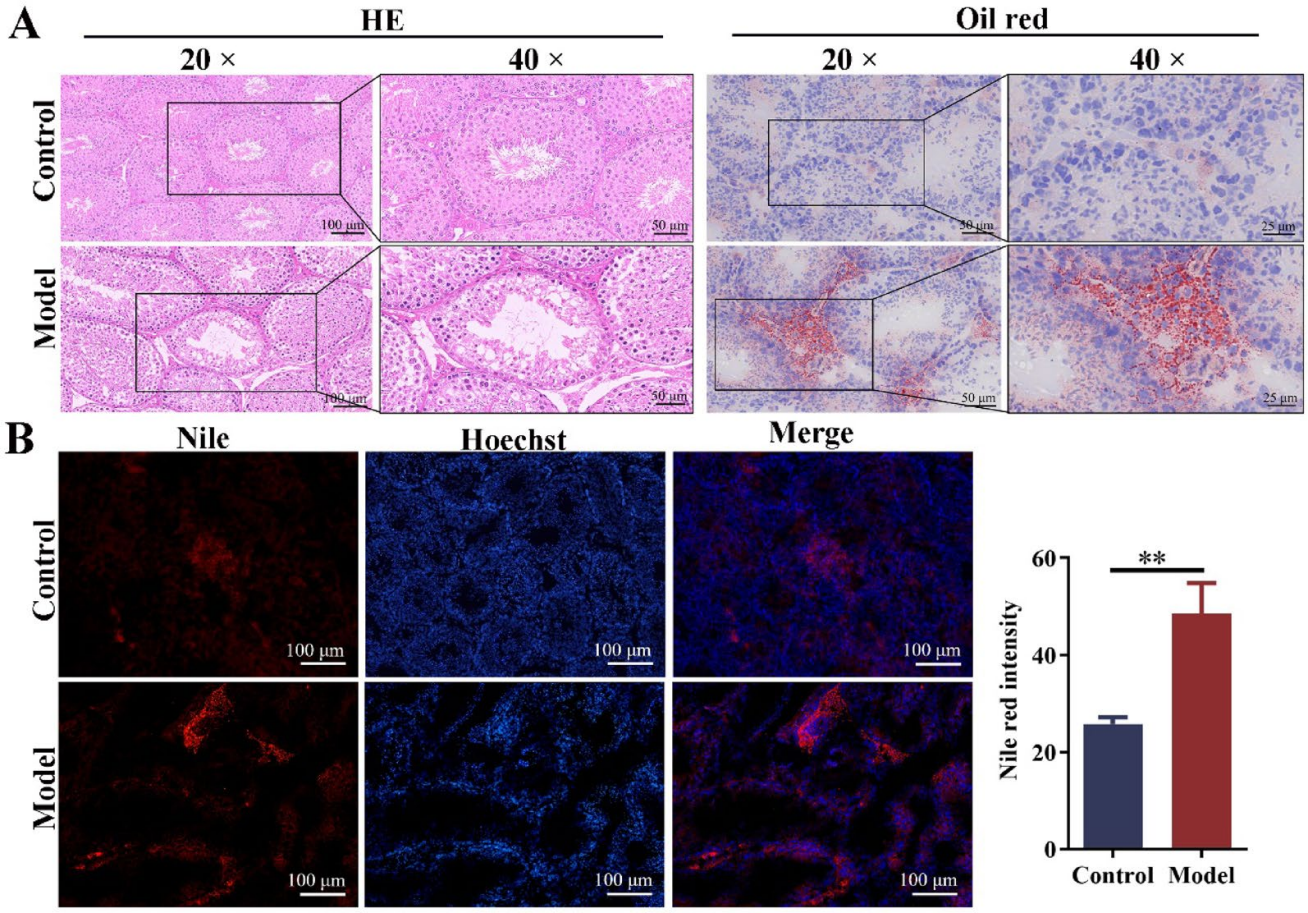
Lipid deposition in the testes of obese mice with oligoasthenospermia. **A** HE and oil red O staining of the testis (scale bar: 100 μm, 50 μm and 25 μm). **B** Nile red staining of the testis (scale bar: 100 μm). n = 3. Compared with the control group, **p < 0.01

In the control group, the seminiferous tubules in the testis were intact, the spermatogenic cells were arranged in order, and there were many sperm in the lumen. In contrast, the spermatogenic cells in the testis of obese mice were loosely arranged and had fewer layers, the sperm in the lumen was significantly reduced, and the Leydig cells and some structures in seminiferous tubules were vacuolated (Fig. 2A). Oil red O and Nile red staining showed that obvious lipid droplets accumulated in the testis of obese mice (Fig. 2A, B). Meanwhile, quantitative analysis revealed that the fluorescence intensity of Nile red was significantly increased in obese mice (Fig. 2B). These results indicated that obesity-induced lipid droplet deposition in the testis of mice.

### The dysregulation of testicular cholesterol metabolism in obese mice with oligoasthenospermia

**Fig. 3:**
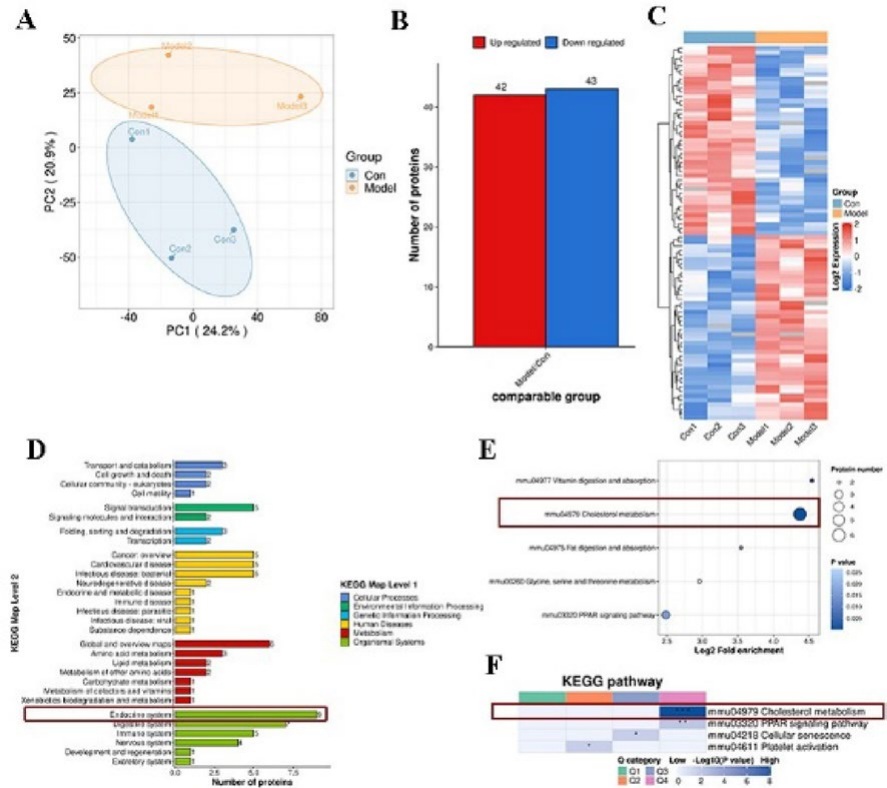
The proteomic analysis of the testis. **A** PCA principal component analysis diagram. **B** Differential protein statistical diagram. **C** Differential protein thermal diagram. **D** KEGG function analysis. **E** KEGG enrichment analysis. **F** KEGG cluster analysis. n = 3

Proteomic analysis revealed significant differences in the principal component analysis of testicular tissue proteins between the two groups (Fig. 3A). Compared with the control group, 42 proteins were significantly upregulated, and 43 proteins were significantly downregulated in the testis of obese mice (using a threshold of 1.3) (Fig. 3B, C). KEGG functional analysis revealed nine differentially expressed proteins related to the endocrine system (Fig. 3D). Both enrichment and clustering analyses of KEGG pathways indicated that the differentially expressed proteins were primarily enriched in cholesterol metabolism pathways (Fig. 3E, F). Therefore, we speculated that obesity-induced abnormal cholesterol metabolism in the testis, thereby causing lipid homeostasis imbalance.

### The expression of HSL was downregulated in the testes of obese mice with oligoasthenospermia

**Fig. 4:**
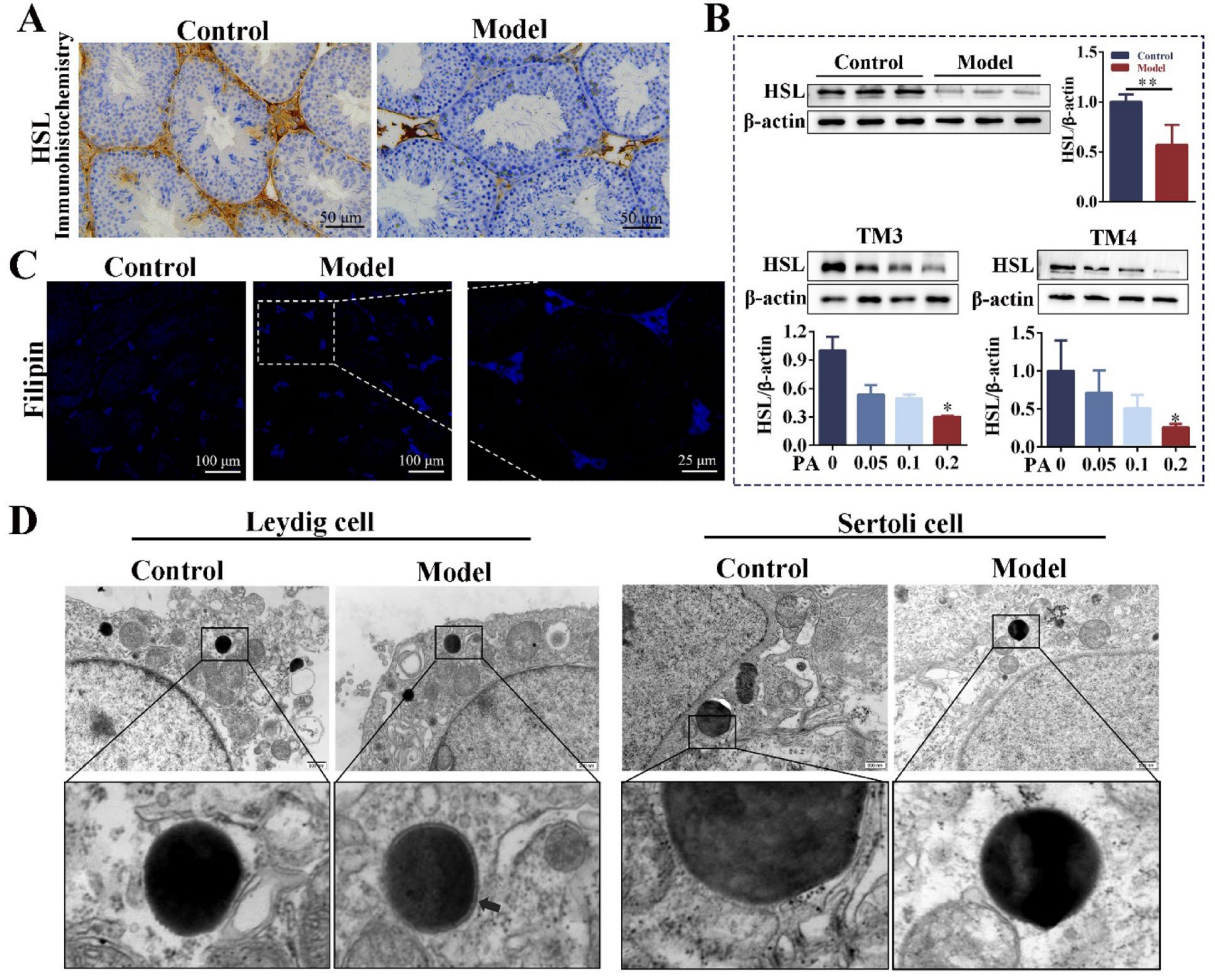
The expression of HSL was downregulated in the testes of obese mice with oligoasthenospermia. **A** Immunohistochemistry was used to detect the distribution and expression of HSL in testis (scale: 50 μm). n = 3. **B** The protein expression of HSL in testis and cells (cells were stimulated with different concentrations of PA for 24 h). n = 6. **C** Filipin staining of testis. n = 3. **D** TEM images in the testicular Leydig and Sertoli cells. PA = palmitic acid, TEM = transmission electron microscope. The arrowhead indicates the membrane of the annular junctions around the lipid droplet is free cholesterol-positive. Compared with the control group, **p < 0.01. Compared with the PA = 0 mM group, *p < 0.05

Immunohistochemistry showed that HSL was highly expressed in Leydig and Sertoli cells of the control group, and the expression of HSL in the testis of obese mice was significantly down-regulated (Fig. 4A). WB results showed that the protein expression of HSL was significantly down-regulated in the testis of the model group. After TM3 cells (Leydig cells) and TM4 cells (Sertoli cells) were stimulated with PA for 24 h, the expression of HSL was gradually down-regulated with the increase of PA concentration (Fig. 4B). Filipin staining of free cholesterol in the testis showed that FC increased in the Leydig cells of obese mice (Fig. 4C). TEM showed that there was a circular connecting membrane composed of FC-positive (Arrows) around the LDs in the Leydig cells of the testis of obese mice (Pelletier 2011). However, no accumulation of free cholesterol was observed at the edge of LDs in Sertoli cells (Fig. 4D). These results indicated that obesity led to LDs deposition and down-regulation of HSL expression in the testis, as well as the increase of FC in Leydig cells. Therefore, we speculated that the key factor of lipid droplet deposition in the testis may be the down-regulation of HSL expression, and the difference of FC in Leydig cells and Sertoli cells may be related to the uptake and efflux of cholesterol.

### Cholesterol uptake and efflux were disrupted in the testes of obese mice with oligoasthenospermia

**Fig. 5:**
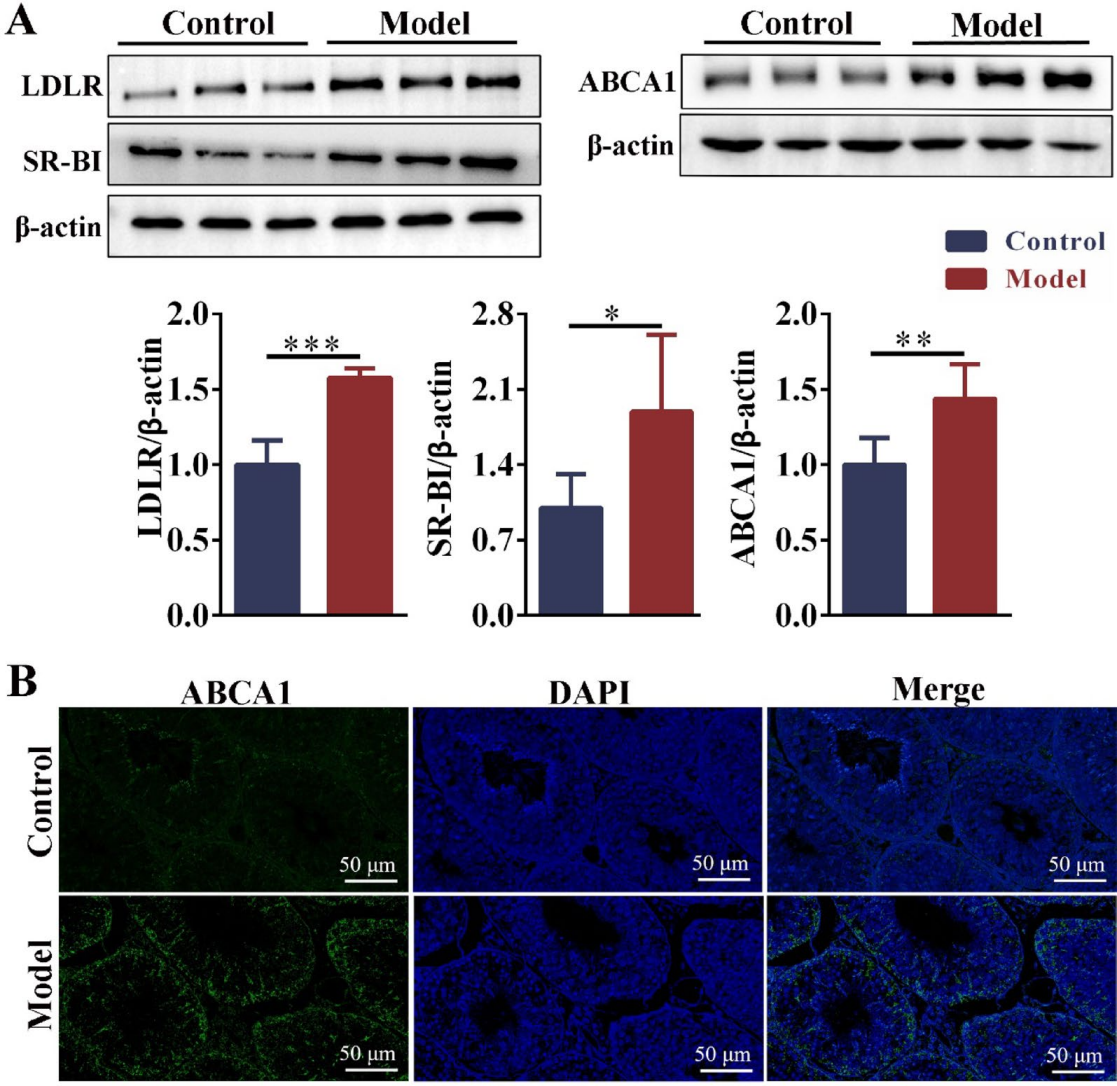
The expression of cholesterol uptake receptors and efflux transporter in testis. **A** The protein expressions of LDLR, SR-BI, and ABCA1 in the testis. n = 6. **B** The distribution of ABCA1 in the testis was observed by immunofluorescence (scale bar: 100 μm). n = 3. Compared with the control group, *p < 0.05, **p < 0.01, ***p < 0.001

Compared with the control group, the expression of LDLR and SR-BI was significantly upregulated in the testis of obese mice. In the model group, the expression of ABCA1 was significantly upregulated (Fig. 5A). Immunofluorescence showed that ABCA1 was upregulated in the Sertoli cells of testis (Fig. 5A, B).

**Fig. 6:**
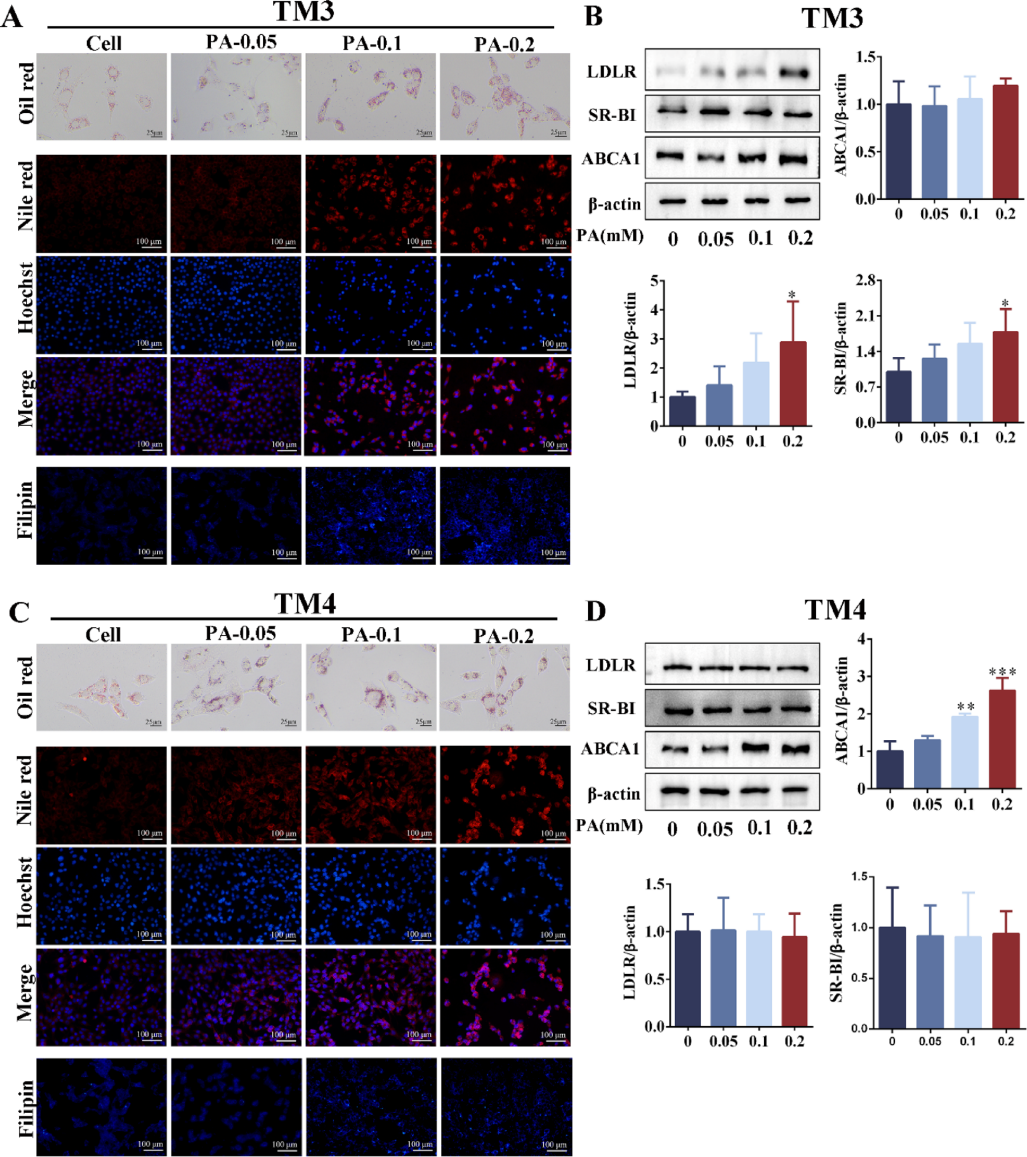
The PA induces disorders of lipid homeostasis and cholesterol metabolism in Leydig and Sertoli cells. **A** The oil red O, Nile red and Filipin staining of TM3 cells after stimulating with PA for 24 h (scale: 25 μm and 100 μm). n = 3. **B** The protein expression levels of LDLR, SR-BI and ABCA1 in TM3 cells after stimulating with PA for 24 h. n = 3. **C** The oil red O, Nile red and Filipin staining of TM4 cells after stimulating with PA for 24 h (scale: 25 μm and 100 μm). n = 3. **D** The protein expression levels of LDLR, SR-BI and ABCA1 in TM4 cells after stimulating with PA for 24 h. n = 3. PA = palmitic acid. TM3 cells (mouse Leydig cells). TM4 cells (mouse Sertoli cells). Compared with the PA = 0 mM group, *p < 0.05, **p < 0.01, ***p < 0.001

TM3 cells and TM4 cells were stimulated with PA, and oil red O and Nile red staining showed that intracellular lipid droplet deposition was aggravated with PA concentration (Fig. 6A, C). Consistent with the in vivo experiments, FC was also expressed differently in TM3 and TM4 cells. The expression of FC was enhanced with increasing PA concentration in TM3, and the expression of FC was attenuated with increasing PA concentration in TM4 (Fig. 6A, C). In TM3 cells, LDLR and SR-BI protein expressions were significantly upregulated after PA stimulation. At the same time, there was no significant difference in ABCA1 expression (Fig. 6B). In TM4 cells, there was no significant difference in the expression of LDLR and SR-BI after PA stimulation. In contrast, the expression of ABCA1 was significantly upregulated (Fig. 6D). We speculated that the expression of SR-BI and LDLR were compensably upregulated to promote cholesterol uptake due to the high demand for cholesterol synthesis steroid hormones in Leydig cells and the inhibition of cholesterol ester hydrolysis (the expression of HSL was down-regulated, Fig. 4A, B). The increased FC in Leydig cells resulted from increased cholesterol uptake (see Figs. 4C, 6A). However, the up-regulation of efflux transporter (ABCA1) mediated the reduction of free cholesterol in Sertoli cells. These results indicated that obesity-induced LDs deposition in both Leydig cells and Sertoli cells in the mouse testes. However, the difference in FC levels between Leydig cells and Sertoli cells may be associated with the upregulation of cholesterol uptake receptors in Leydig cells and the upregulation of efflux transporters in Sertoli cells.

### Testosterone synthesis was impaired in obese mice with oligoasthenospermia

**Fig. 7:**
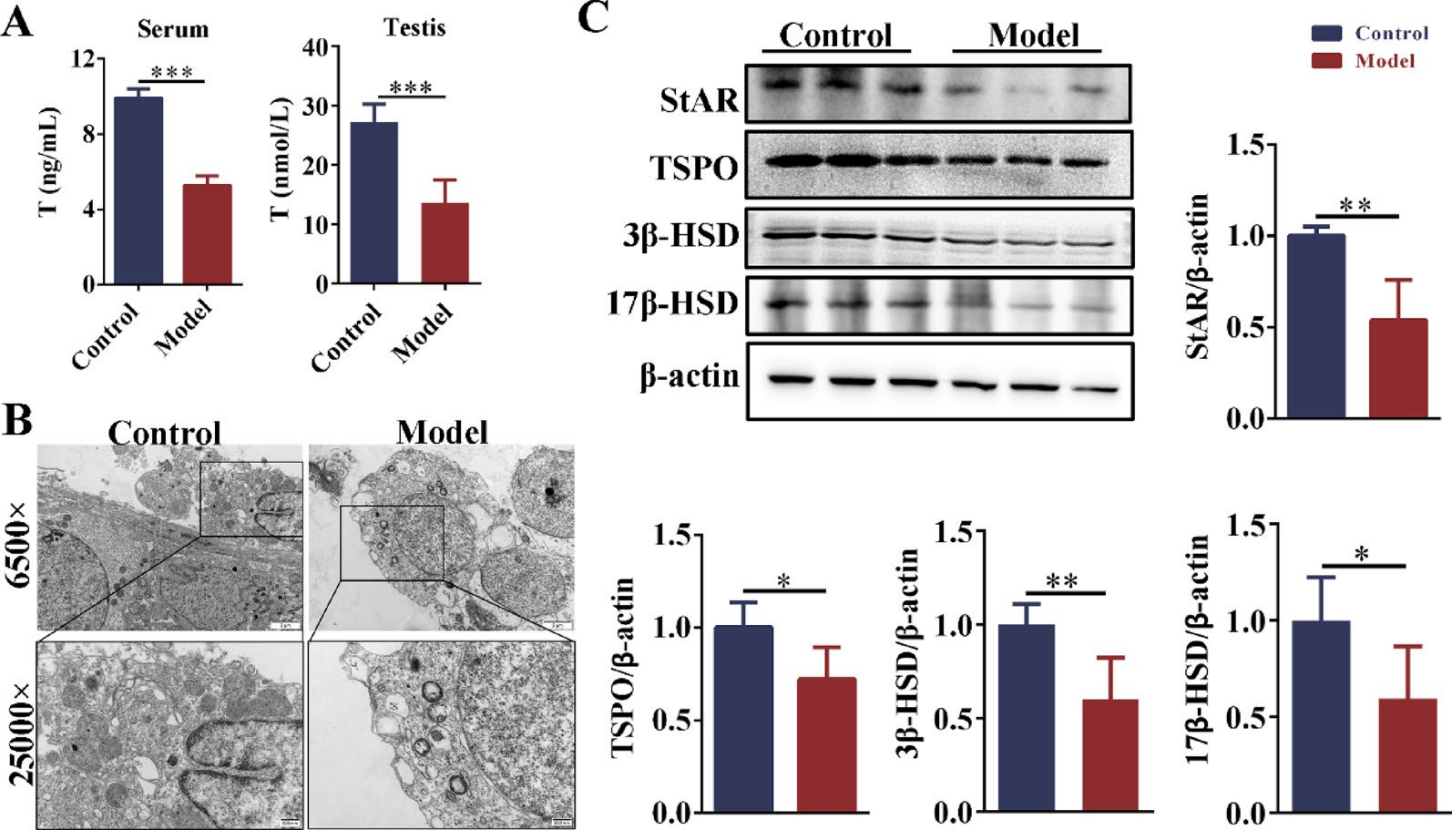
Testosterone synthesis disorder in obese mice with oligoasthenospermia. **A** The levels of testosterone in serum and testis. n = 6. **B** The mitochondrial structure in Leydig cells was observed via TEM (magnification: × 6,500 and × 25,000). n = 3. **C** The protein bands and analysis of the testosterone synthesis-related enzymes (StAR, TSPO, 3β-HSD and 17β-HSD). n = 6. T = testosterone. Compared with the control group, *p < 0.05, **p < 0.01, ***p < 0.0013

Compared with the control group, serum and testicular testosterone levels were significantly reduced in the model group (Fig. 7A). In testosterone synthesis, cholesterol is first transported to mitochondria. TEM was used to observe the structure of mitochondria in Leydig cells. In the control group, the mitochondrial structure was intact, and the cristae were clearly visible and arranged in an orderly manner. In contrast, the model group showed a large number of mitochondrial damages, including morphological swelling, cristae rupture, matrix inhomogeneity, and vacuolization (Fig. 7B). The mitochondrial outer membrane protein TSPO facilitates cholesterol transport to the inner mitochondrial membrane through its interaction with StAR. Western blot analysis revealed that the expression of StAR and TSPO in the testes of the model group was significantly downregulated compared to the control group. Cholesterol is converted to pregnenolone in the mitochondria, which is then transported to the endoplasmic reticulum and further metabolised into testosterone by dehydrogenases. The results showed that the expressions of 3β-HSD and 17β-HSD in the testes of the model group were significantly down-regulated (Fig. 7C). These results indicated that LDs deposition in testis led to mitochondrial structural damage, down-regulation of steroid hormone synthase expression, and then testosterone synthesis disorder.

### The blood-testis barrier connections were impaired in obese mice with oligoasthenospermia

**Fig. 8:**
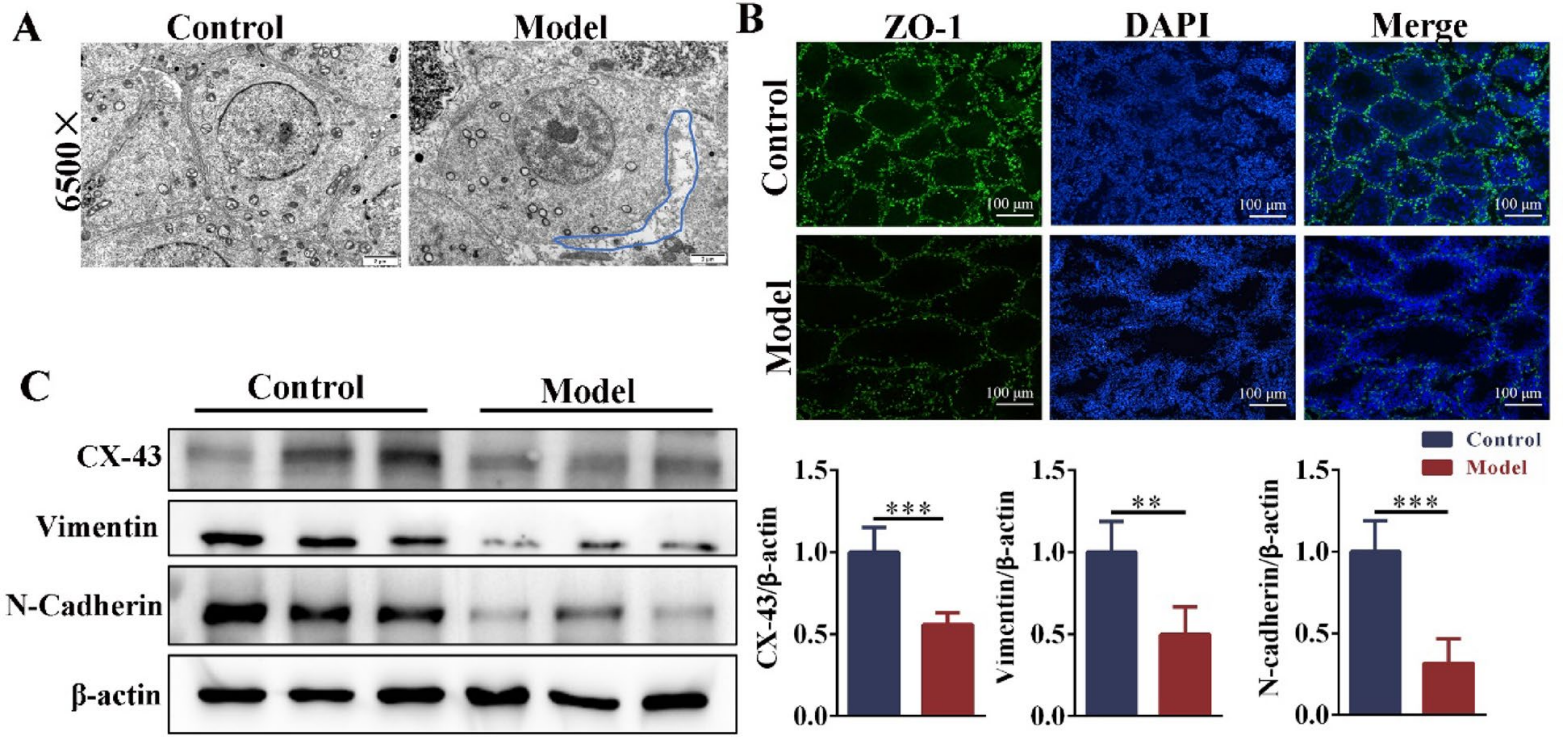
The BTB integrity was damaged in obese mice with oligoasthenospermia. **A** The intercellular junction in the testis was observed via TEM (magnification: × 6500). n = 3. **B** The immunofluorescence expression of ZO-1 in the testis (scale bar: 100 μm). n = 3. **C** The protein expressions of BTB junction (Cx-43, vimentin, and N-cadherin). n = 6. BTB = blood‒testis barrier. Compared with the control group, **p < 0.01, ***p < 0.001

Sertoli cells, which are somatic cells in close contact with germ cells, provide nutritional and structural support for developing spermatogenic cells. TEM showed that the intercellular junctions in the testis of control mice were tight and orderly, and the junction between germ cells and Sertoli cells in obese mice was damaged. The intercellular space was enlarged (Fig. 8A). Sertoli cells form the BTB through tight junctions, preventing harmful substances from entering the seminiferous tubules. In the model group, the fluorescence expression of ZO-1 was reduced in the testes (Fig. 8B), and the expressions of BTB junction-related proteins (Vimentin, N-Cadherin, and Cx-43) were significantly down-regulated (Fig. 8C, D). These results indicated that obesity led to LDs deposition and the reduction of FC in Sertoli cells, which impaired the testicular BTB junction.

### Overexpression of HSL reduced lipid deposition and increased testosterone levels in Leydig cells

**Fig. 9:**
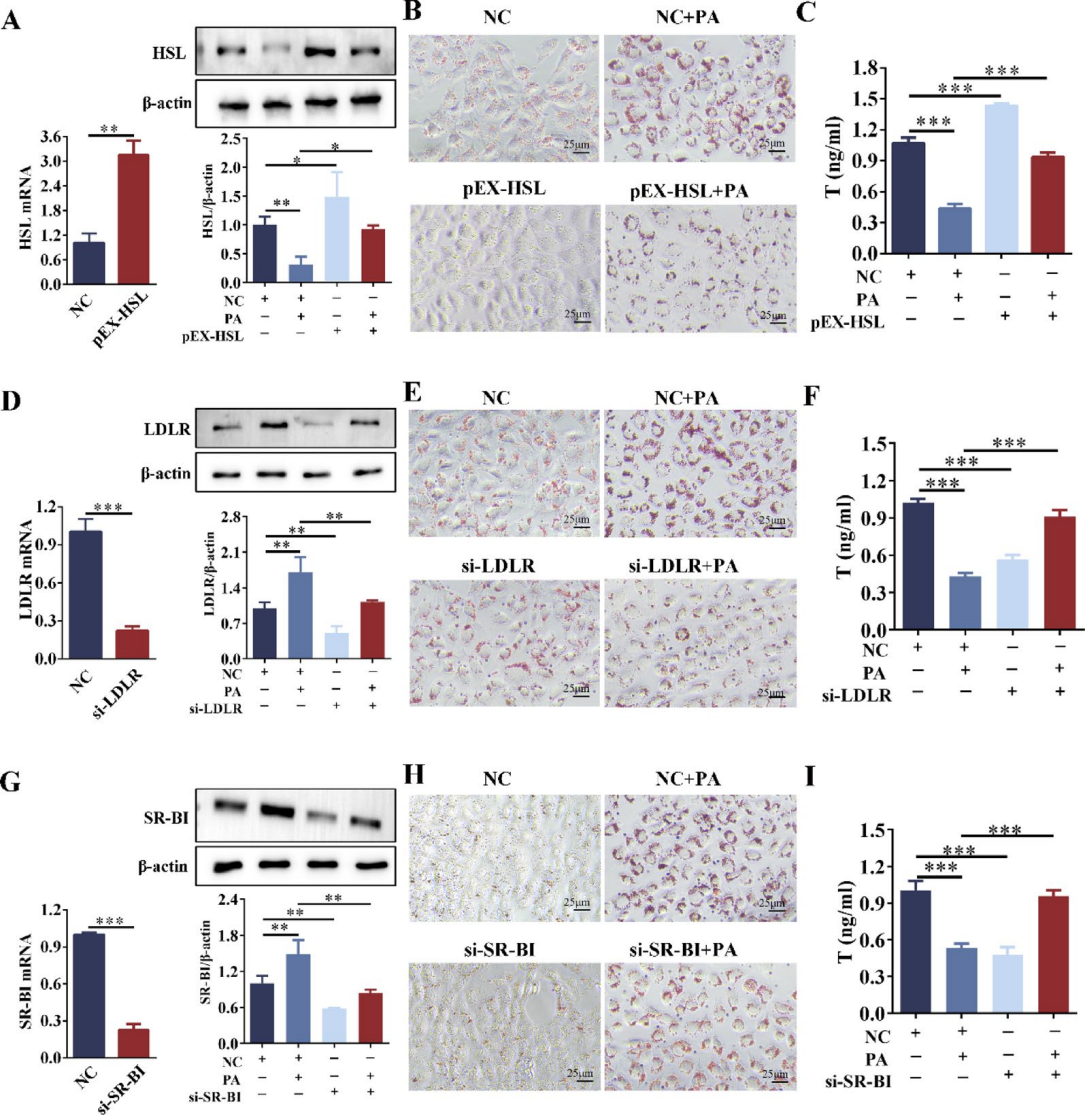
Effects of overexpressing HSL or silencing LDLr/SR-BI on lipid homeostasis and testosterone in TM3 cells. **A** qRT-PCR was used to detect the overexpression efficiency of HSL in TM3 cells. TM3 cells were stimulated with PA (0.2 mM) for 24 h after pEX-HSL transfection, and the protein expression of HSL was detected by Western blot. n = 3. **B** The Oil red O staining of TM3 cells after pEX-HSL transfection or/and PA stimulation. n = 3. **C** Testosterone level of TM3 cells after pEX-HSL transfection or/and PA stimulation. n = 3. **D** qRT-PCR detected the silencing efficiency of LDLR in TM3 cells. TM3 cells were stimulated with PA (0.2 mM) for 24 h after si-LDLR transfection, and LDLR expression was detected by Western blot. n = 3. **E** The Oil red O staining of TM3 cells after si-LDLR transfection or/and PA stimulation. n = 3. **F** Testosterone level of TM3 cells after si-LDLR transfection or/and PA stimulation. n = 3. **G** qRT-PCR detected the silencing efficiency of SR-BI in TM3 cells. TM3 cells were stimulated with PA (0.2 mM) for 24 h after si-SR-BI transfection, and SR-BI expression was detected by Western blot. n = 3. **H** The Oil red O staining of TM3 cells after si-SR-BI transfection or/and PA stimulation. n = 3. **I** Testosterone level of TM3 cells after si-SR-BI transfection or/and PA stimulation. n = 3. NC = Negative control. *p < 0.05, **p < 0.01, ***p < 0.001

Based on the results from Figs. 4, 5, 6, and 7, we concluded that the downregulation of HSL expression and the compensatory upregulation of LDLR and SR-BI in Leydig cells led to excessive intracellular LDs accumulation and FC retention, thereby impairing testosterone synthesis. To investigate whether targeting these molecules could improve lipid deposition and testosterone secretion, reverse interventions were performed on these targets. Overexpression of HSL: Compared with the NC group, the mRNA and protein levels of HSL were significantly upregulated after HSL overexpression (Fig. 9A). Compared with the PA group, pEX-HSL reversed the inhibitory effect of PA on HSL expression, significantly upregulated HSL expression, and reduced LDs deposition (Fig. 9A, B). Silencing LDLr: Compared with the NC group, the mRNA and protein levels of LDLr were significantly downregulated in the si-LDLr group (Fig. 9E). Compared with the PA group, the si-LDLr + PA group showed down-regulated LDLr expression and decreased LDs deposition (Fig. 9E, F). Silencing SR-BI: Compared with the NC group, the mRNA and protein levels of SR-BI were significantly down-regulated in the si-SR-BI group (Fig. 9G). Compared with the PA group, the si-SR-BI + PA group exhibited significantly down-regulated SR-BI expression and decreased LDs deposition (Fig. 9G, H). In summary, overexpression of HSL or knockdown of LDLR/SR-BI all reduced LDs deposition in Leydig cells.

Compared with the NC group, PA stimulation significantly reduced testosterone levels. In contrast, the pEX-HSL + PA, si-LDLR + PA, and si-SR-BI + PA groups exhibited significantly increased testosterone levels compared to the PA group (Fig. 9C, F, I). Compared with the NC group, pEX-HSL promoted testosterone production. However, the knockdown of LDLR or SR-BI inhibited testosterone production (Fig. 9F, I). Therefore, the low expression of LDLR/SR-BI had a negative impact on testosterone synthesis in Leydig cells.

### Overexpression of HSL reduced lipid deposition in Sertoli cells

**Fig. 10:**
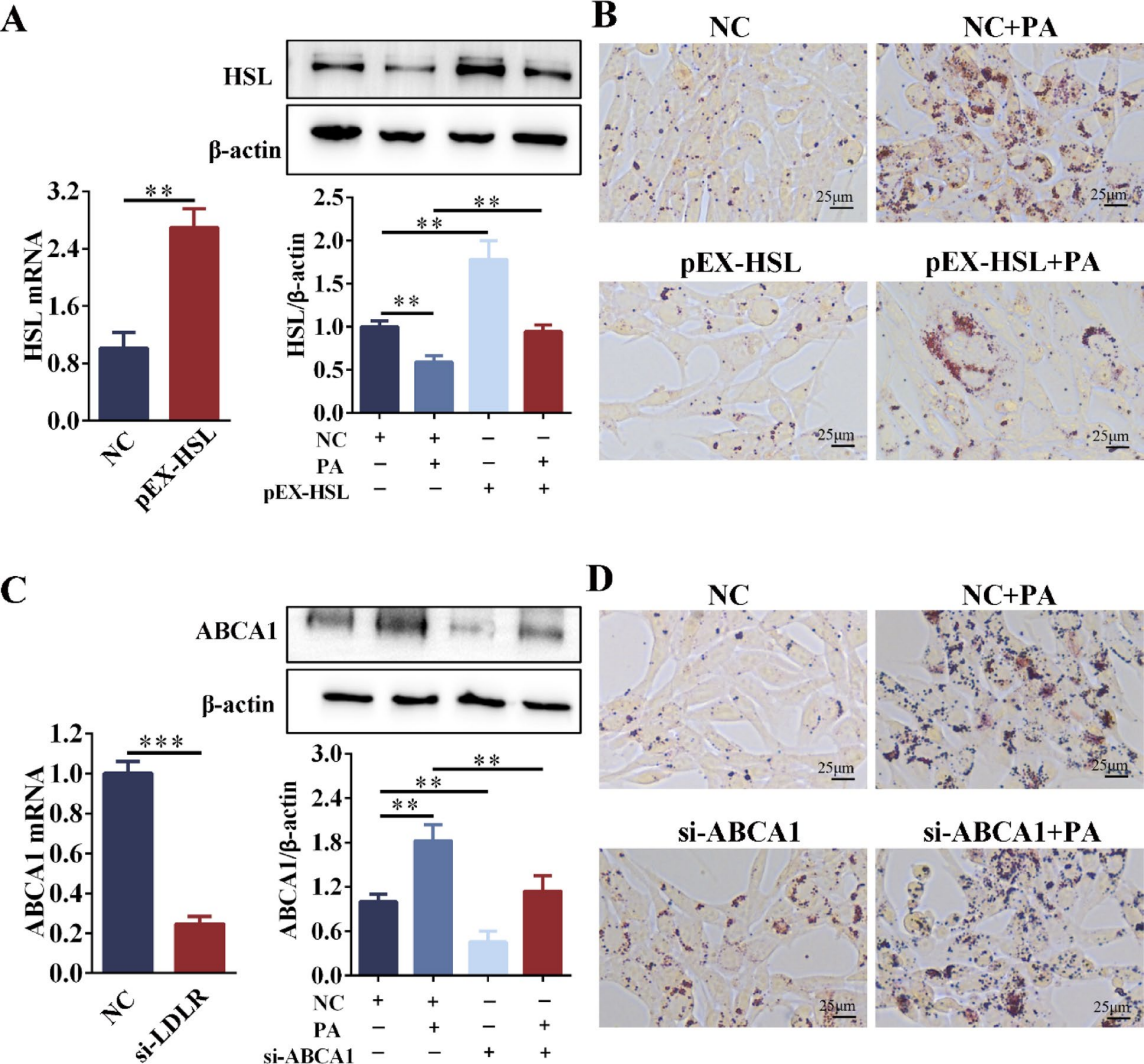
Effects of overexpressing HSL or silencing ABCA1 on lipid homeostasis in TM4 cells. **A** qRT-PCR was used to detect the overexpression efficiency of HSL in TM4 cells. TM4 cells were stimulated with PA (0.2 mM) for 24 h after pEX-HSL transfection, and the expression of HSL was detected by Western blot. n = 3. **B** The Oil red O staining of TM4 cells after pEX-HSL transfection or/and PA stimulation. n = 3. **C** qRT-PCR detected the silencing efficiency of ABCA1 in TM4 cells. TM4 cells were stimulated with PA (0.2 mM) for 24 h after si-ABCA1 transfection, and ABCA1 expression was detected by Western blot. n = 3. **D** The Oil red O staining of TM3 cells after si-ABCA1 transfection or/and PA stimulation. n = 3. n = 3. NC = Negative control. **p < 0.01

To determine whether modulation of these molecules could ameliorate LDs accumulation in Sertoli cells, we performed reverse intervention experiments targeting these factors in TM4 cells. Overexpression of HSL: Compared with the NC group, the mRNA and protein levels of HSL were significantly upregulated after HSL overexpression (Fig. 10A). Compared with the PA group, pEX-HSL reversed the inhibitory effect of PA on HSL expression, significantly up-regulated HSL expression, and reduced intracellular lipid droplet deposition (Fig. 10A, B). Silencing ABCA1: Compared with the NC group, the mRNA and protein levels of ABCA1 were significantly downregulated in the si-ABCA1 group, and the low expression of ABCA1 resulted in increased intracellular lipid droplets (Fig. 10C). Compared with the PA group, the si-ABCA1 + PA group showed significantly down-regulated ABCA1 expression but no corresponding reduction LDs deposition (Fig. 10C, D). Thus, overexpression of HSL reduced LDs deposition in Sertoli cells.

## Discussion

With the rapid increase in the incidence of obesity, obesity-induced oligoasthenospermia has become the leading cause for male infertility. Several studies have shown that HFD can cause testicular damage, decreased semen quality, and gonadal axis disorders (Darand et al. 2023; George et al. 2023). The body weight and blood lipid levels of obese mice increased significantly. Interestingly, there was no significant difference in food intake between the two groups. Although there was no difference in food intake between the two groups, the model group fed with HFD (60% fat) actually consumed much higher total calories, resulting in excess energy being converted into fat and stored. Meanwhile, obese mice exhibited significantly elevated serum leptin levels (*p* < 0.001). We speculated that this may affect the appetite of mice and inhibit excessive intake. With the increase in body weight, the testis and epididymis coefficients decreased, sperm quality declined, and hormone levels became disordered. This suggested that obesity was closely associated with oligoasthenospermia.

In the testis, lipid balance is crucial to maintaining normal reproductive function. This study showed that obvious LDs were deposited in the testis of HFD-induced obese mice. Meanwhile, testicular proteomics showed that abnormal cholesterol metabolism in the testis of obese mice. These findings indicated that HFD led to a disorder of cholesterol metabolism in the mouse testis, which in turn disrupted intracellular lipid homeostasis. Cholesterol is an important substrate for androgen synthesis, and it is the key material to maintain the structure and function of cell membrane and regulate cell proliferation (Schade et al. 2020). Cholesterol metabolism plays a crucial role in the male reproductive system, and its dysregulation may lead to impaired spermatogenesis, disrupted hormone synthesis, and diminished reproductive function. (De Toni et al. 2021; Cross 1998). The clinical study found that the expression of HSL in the sperm of obese patients was inhibited (Calderón et al. 2019). Studies have shown that inhibting HSL in the testis increases intracellular LDs (Casado et al. 2021). In this study, the expression of HSL was down-regulated in testicular Leydig cells and Sertoli cells of HFD mice, which led to LDs deposited in the testis and cells.

In obese mouse testes and PA-induced cells, the expression of FC was inconsistent, which should be related to cholesterol uptake receptors and efflux transporters. In autoimmune orchitis, the increase of SR-BI expression was positively correlated with cholesterol ester levels (Akpovi et al. 2006). Casado et al. (2012) found that the expression of SR-BI was increased in the testis of HSL^−/−^ mice, which destroyed the cholesterol homeostasis in the testis. Calderón et al. (2019) reported that when the expression of HSL disappeared in the sperm of obese patients, the expression of LDLr increased with compensation. It was reported that the increase of SR-BI and LDLr expression may compensate for the decrease of intracellular cholesterol ester hydrolysis caused by HSL inhibition (Casado et al. 2021). This study demonstrated that the expressions of LDLR and SR-BI were significantly increased in testicular Leydig cells of obese mice. On the one hand, the increase of LDL-C content in blood may promote the upregulation of cholesterol uptake receptor expression in Leydig cells. On the other hand, Leydig cells need a large amount of free cholesterol for testosterone synthesis, and the hydrolysis pathway of these intracellular cholesterol esters is inhibited. Therefore, SR-BI and LDLR expression were compensatory upregulated to enhance FC levels in the Leydig cells. In this study, ABCA expression was down-regulated in testicular Sertoli cells of obese mice. When PA-induced lipid deposition in Sertoli cells (TM4 cells), intracellular TC levels increased, while intracellular FC levels decreased (Dong et al. 2024). Our results showed that FC was reduced in Sertoli cells (filipin staining). However, elevated LDL-C levels in the blood did not lead to increased cholesterol uptake receptors, which we speculate may be due to the obstruction of the BTB. Therefore, ABCA1 expression was upregulated to increase intracellular FC in the Sertoli cells.

Clinical data showed that the levels of total testosterone and bioavailable testosterone were decreased in obese men (Wittert and Umapathysivam 2024). Consistent with this, our research results showed that the levels of testosterone also decreased significantly in the serum and TM3 cells (Leydig cells) of obese mice. Yu et al. (2019) found that HFD increased cholesterol accumulation in rat Leydig cells and decreased serum testosterone levels. In Leydig cells, cholesterol is necessary to synthesize testosterone. However, excessive cholesterol accumulation may cause "cholesterol toxicity" and destroy the testosterone synthesis pathway in the testis (Song et al. 2021; Wang et al. 2015). Excessive accumulation of lipid droplets may hurt spermatogenesis by physically interfering with the normal structure and function of mitochondria (Vertika et al. 2020). Our results showed that the expressions of steroid hormone synthesis-related enzymes (StAR, TSPO, 17β-HSD, and 3β-HSD) were significantly down-regulated in the testis of obese mice. Studies have shown that the activity of StAR is affected by HSL. When HSL is inhibited, the activity of StAR is decreased (Zirkin and Papadopoulos 2018). The key role of StAR in cholesterol transport and steroid hormone synthesis has been widely confirmed.For example, testosterone was significantly decreased in star knockout (KO) mice and star knockout MA-10 cells (tumor stromal cells in mice) (Galano et al. 2021). In summary, our findings demonstrated that HFD suppressed the expression of HSL in testicular Leydig cells. Concurrently, HFD upregulated the expression of LDLr and SR-BI, leading to lipid accumulation in Leydig cells. This lipid deposition subsequently disruptds mitochondrial integrity and downregulated key enzymes involved in testosterone synthesis, ultimately resulting in reduced testosterone production.

Sertoli cells form tight intercellular junctions that establish the BTB, which is essential for providing structural support and nutritional regulation to developing spermatogenic cells. Studies have demonstrated that HFD compromises BTB integrity in murine testicular tissue, ultimately contributing to impaired fertility (Wei et al. 2023; Zhang et al. 2023). Using TEM and immunofluorescence, we observed an abnormal widening of the intercellular space between spermatogenic cells and Sertoli cells in the testes of HFD-induced obese mice, along with structural disruption of the BTB. Yang et al. (2022) reported that the accumulation of lipid ROS would destroy the integrity of BTB in mouse Sertoli cells. Furthermore, excessive intracellular lipid accumulation promoted lipid peroxidation, thereby compromising cellular structure and function. (Gaschler and Stockwell 2017). Our study revealed significant lipid accumulation in Sertoli cells of HFD-induced obese mice, likely attributable to the downregulation of HSL expression. The reduced HSL activity impaired cholesterol ester hydrolysis, resulting in intracellular LD accumulation, which may contribute to oxidative damage to the BTB. Concurrently, we observed a marked upregulation of ABCA1 in Sertoli cells of obese mice, which enhanced FC efflux. This elevated cholesterol export potentially depleted the cellular pool of free cholesterol available for membrane biosynthesis, further compromising BTB integrity. These findings demonstrate that HFD-induced lipid deposition and free cholesterol depletion in Sertoli cells destabilized their cellular membranes, ultimately disrupting intercellular junctions and impairing BTB function.

To investigate whether modulation of HSL and cholesterol receptors/transporters could ameliorate intracellular lipid accumulation, we performed transfection experiments in PA-treated Leydig cells. Ectopic expression of HSL or genetic ablation of either LDLr or SR-BI significantly attenuated lipid droplet accumulation and restored testosterone production in PA-exposed cells. Notably, while knockdown of LDLr or SR-BI in untreated control cells similarly reduced lipid deposition, this intervention failed to enhance testosterone synthesis, suggesting distinct regulatory mechanisms governing lipid homeostasis and steroidogenesis Studies showed that the testosterone levels were decreased in LDLR^*−/−*^ mice (Bai et al. 2023). When the expression of SR-BI was inhibited, the supply of intracellular cholesterol was insufficient, and the synthesis of testosterone was reduced (Gao et al. 2018). Collectively, our findings suggested that upregulated expression of LDLr and SR-BI in Leydig cells promoted both LDs accumulation and FC deposition, ultimately resulting in cholesterol-mediated cytotoxicity. The ABCA1^−/−^ mice showed LD accumulation in Sertoli cells (Selva et al. 2004). Our results also showed that knockdown of ABCA1 resulted in lipid droplet deposition in Sertoli cells. In summary, our findings demonstrated that targeted activation of HSL effectively mitigated testicular lipid accumulation, enhanced testosterone biosynthesis, and promoted spermatogenic recovery in obesity with oligoasthenospermia.

Nevertheless, our study is not without limitations. The testis comprises three major cell types: Leydig cells, Sertoli cells, and spermatogenic cells. While existing evidence confirms that disrupted lipid homeostasis impairs the function of both Leydig and Sertoli cells, thereby indirectly affecting spermatogenesis, whether lipid imbalance directly compromises the development of spermatogenic cells remains to be fully elucidated. HSL is required for spermatogenesis. HSL^−/−^mice also show a dramatic reduction in sperm counts and motility (Hermo et al. 2008). Excessive lipid accumulation within the testes, including cholesterol, triglycerides and specific fatty acids, disrupts essential sperm production processes such as membrane formation, maturation, energy metabolism and cell signalling. This leads to apoptosis, impaired spermatogenesis, and abnormal sperm morphology and function (Saez Lancellotti et al. 2025).

## Conclusion

**Fig. 11:**
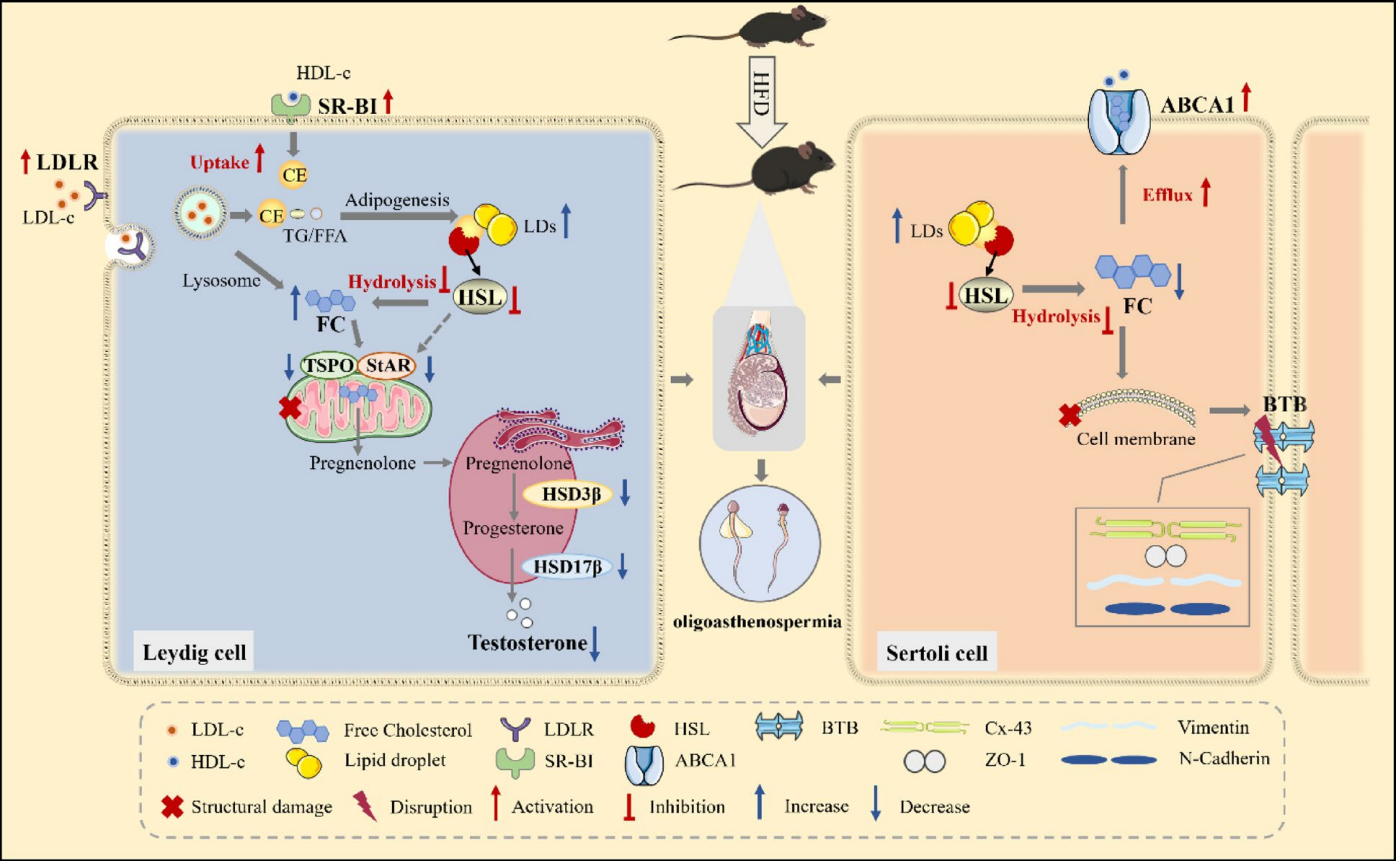
Schematic illustration of this study. The down-regulation of hormone-sensitive lipase and the dysregulation of cholesterol receptor/transporter destroy the lipid homeostasis of testis in mice, which leads to the disorder of testosterone synthesis and BTB integrity, thus affecting the process of spermatogenesis

In summary, HFD-induced obesity disrupts testicular lipid homeostasis through dual cellular mechanisms, ultimately driving oligoasthenospermia. In Leydig cells, HFD suppressed HSL expression while upregulating LDLr and SR-BI, resulting in pathological accumulation of LDs and FC. This lipid overload-induced mitochondrial structural damage and downregulated steroidogenic enzymes, culminating in impaired testosterone biosynthesis. Meanwhile, HFD perturbed Sertoli cell function via HSL suppression and ABCA1 upregulation, triggering aberrant LD deposition alongside FC depletion. These perturbations compromised BTB integrity. Therefore, obesity disrupted the lipid metabolism of both Leydig cells and Sertoli cells, delivering a “double blow” to spermatogenesis by impairing hormone synthesis and structural integrity, eventually resulting in oligoasthenospermia (Fig. 11).

## Electronic supplementary material

Below is the link to the electronic supplementary material.

Supplementary Material 1

Supplementary Material 2

## Data Availability

No datasets were generated or analysed during the current study.
