# Supplementary material for: The downregulation of hormone-sensitive lipase and dysregulation of cholesterol receptors/transporter affect testicular lipid homeostasis and function in HFD-induced oligoasthenospermia mice
Source: Mol Med. 2025 Aug 4;31:274. doi: 10.1186/s10020-025-01327-x (PMC12323199; doi:10.1186/s10020-025-01327-x)
Supplement: Supplementary file 1 — Supplementary Material 1 [file 10020_2025_1327_MOESM1_ESM.docx]

| **Gene** | **Forward Primer (5`-3`)** | **Reverse Primer (5`-3`)** |
| --- | --- | --- |
| ***Scarb1-siRNA*** | GCCUGUUUGUUGGGAUGAATT | UUCAUCCCAACAAACAGGCTT |
| ***Ldlr-siRNA*** | CCGUCUCUAUUGGGUUGAUTT | AUCAACCCAAUAGAGACGGTT |
| ***Abca1-siRNA*** | CCAGCUGAAGGGCUGGAAATT | UUUCCAGCCCUUCAGCUGGTT |

Supplementary Table 1. Silencing gene sequence and overexpressing plasmid vector.

| **Gene** | **Accession number/Gene ID** | **Vector name** |
| --- | --- | --- |
| ***pEX-Hsl*** | >NM_010719.5:379-2787 Mus musculus lipase, hormone sensitive (Lipe), transcript | pcDNA3.1(+) |
