# Supplementary material for: The downregulation of hormone-sensitive lipase and dysregulation of cholesterol receptors/transporter affect testicular lipid homeostasis and function in HFD-induced oligoasthenospermia mice
Source: Mol Med. 2025 Aug 4;31:274. doi: 10.1186/s10020-025-01327-x (PMC12323199; doi:10.1186/s10020-025-01327-x)
Supplement: Supplementary file 2 — Supplementary Material 2 [file 10020_2025_1327_MOESM2_ESM.docx]

|  | **Primer sequence** | |
| --- | --- | --- |
| **Gene** | **Forward** | **Reverse** |
| ***Hsl*** | CTCACAGTTACCATCTCACCTC | GATTTTGCCAGGCTGTTGAGTA |
| ***Scarb1*** | AACATCACCTTCAATGACAACG | ACCAAGATGTTAGGCAGTACAA |
| ***Ldlr*** | CAGAAGTCGACACTGTACTGAC | AAGATGGACAGGAACCTCATAC |
| ***Abca1*** | CCTCAGAGAAAACAGAAAACCG | CTTTGCTATGATCTGCACGTAC |
| ***β-actin*** | ACCGAAGCTCCAATGAATCC | CCGGTGGTTCTACCAGAAGAG |

Supplementary Table 2. Sequences of primers used for real-time quantitative PCR.
